# Supplementary material for: Purification of Functional Human TRP Channels Recombinantly Produced in Yeast
Source: Cells. 2019 Feb 11;8(2):148. doi: 10.3390/cells8020148 (PMC6406451; doi:10.3390/cells8020148)
Supplement: Supplementary file 1 [file cells-08-00148-s001.pdf]

Supplementary Materials

Supplementary Table 1. Sequences of primers used to obtain recombinant transient receptor potential (TRP) channel constructs included in the study. Purified targets are shown in bold.

Target	Primers 5′- 3′
GFP-TEV_FW	AAAATTTGTATTTTCAAAGTCAATTTTCTAAAGGTGAAGAATTATTCACT
GFP-HIS8_RW	CTTCAATGCTATCATTTCCTTTGATATTGGATCATCTAATGGTGATG GTGATGGTGATGGTGTTTGTACAATTCA
TRPC3_FW	ACACAAATACACACACTAAATTACCGGATCAATTCTAAGATAATTATGTCTACTAAAGTCAGAAAGTGTAAG
TRPC3_RW	AAATTGACTTTGAAAATACAAATTTTCTTCACATCTTAACATTGATGGGTTTAAC
**TRPC4_FW**	ACACAAATACACACACTAAATTACCGGATCAATTCTAAGATAATTATGGCACAATTTTATTACAAGAG-
**TRPC4_RW**	AAATTGACTTTGAAAATACAAATTTTCCAATCTTGTAGTGACGTAGTC
TRPC5_FW	ACACAAATACACACACTAAATTACCGGATCAATTCTAAGATAATTATGGCACAATTGTACTACAAGAAGG
TRPC5_RW	AAATTGACTTTGAAAATACAAATTTTCCAATCTTGTAGTAACTTGTTCTTCTTG
TRPV1_FW	ACACAAATACACACACTAAATTACCGGATCAATTCTAAGATAATTATGAAAAAATGGAGTAGTACCG
TRPV1_RW	AAATTGACTTTGAAAATACAAATTTTCCTTTTCACCACTAGCGGCTG
**TRPV3_FW**	ACACAAATACACACACTAAATTACCGGATCAATTCTAAGATAATTATGAAGGCTCACCCTAAAGAAATG
**TRPV3_RW**	AAATTGACTTTGAAAATACAAATTTTCAACACTAGTTTCTGGAAATTCTTC
TRPV4_FW	ACACAAATACACACACTAAATTACCGGATCAATTCTAAGATAATTATGGCTGATTCTTCAGAAGGTC
TRPV4_RW	AAATTGACTTTGAAAATACAAATTTTCCAATGGAGCGTCATCAGTTCTC
TRPML1_FW	ACACAAATACACACACTAAATTACCGGATCAATTCTAAGATAATTATG ACAGCACCAGCAGGTC
TRPML1_RW	AAATTGACTTTGAAAATACAAATTTTCATTAACTAACAATGAATGTTCTTC
**TRPML2_FW**	ACACAAATACACACACTAAATTACCGGATCAATTCTAAGATAATTATG GCAAGACAACCTTACAGATTC
**TRPML2_RW**	AAATTGACTTTGAAAATACAAATTTTCACTGATAGGTATCAAGTGGTC
TRPML3_FW	ACACAAATACACACACTAAATTACCGGATCAATTCTAAGATAATTATGGCAGATCCAGAAGTAGTAG

TRPML3_RW	AAATTGACTTTGAAAATACAAATTTTCCTTTTTGCAACAGCAAAATAAAG
TRPM1_FW	ACACAAATACACACACTAAATTACCGGATCAATTCTAAGATAATTATGTCTTCATTTAAAAGAGGTTCCTTG
TRPM1_RW	AAATTGACTTTGAAAATACAAATTTTCACATTCTGTTTCAGTTGATGCTTTTTC
**TRPM8_FW**	ACACAAATACACACACTAAATTACCGGATCAATTCTAAGATAATTATGAGTTTTAGAGCTGCAAGATTGTC
**TRPM8_RW**	AAATTGACTTTGAAAATACAAATTTTCCTTTATTTTATTAGCAATTTCCTTCAACAAAC

GFP: green fluorescent protein

TEV: Tobacco Etch Virus protease cleavage site

Supplementary Table 2. Solubilization of selected human TRP channels. Two zwitterionic detergents (FC-12 and FC-16) and three non-ionic detergents (DDM, DM and LMNG) were used in solubilization screen. Solubilization efficacies (%) in FC-12 (at a concentration of 1 %), FC-16 (1 %), DDM (2 %), DM (2 %) and LMNG (2%) of crude *S. cerevisiae* membranes for selected TRP channels are shown. Solubilization was performed for 2 h at 4 °C and GFP fluorescence of the solubilized material (supernatant following ultracentrifugation) was used to calculate the percentage of extraction.

Target	Detergent	Concentration, %	Solubilization efficacy, %
TRPC4	FC-12	1	98,73
	FC-16	1	64,91
	DDM	2	43,40
	LMNG	2	29,09
TRPV3	FC-12	1	96,76
	FC-16	1	74,91
	DM	2	43,40
	LMNG	2	29,09
TRPML2	FC-12	1	85,09
	FC-16	1	47,33
	DDM	2	18,26
	LMNG	2	11,28
TRPM8	FC-12	1	95,09
	FC-16	1	84,73
	DDM	2	20,26
	LMNG	2	11,28
